# Cervical Cancer Screening Rates and Associated Sociodemographic and Behavioral Factors among People Experiencing Homelessness in Indiana

**DOI:** 10.21203/rs.3.rs-8524257/v1

**Published:** 2026-01-30

**Authors:** Natalia M. Rodriguez, Xue Case, Lara Balian, Rebecca Ziolkowski, Kalesia Smith, Janelle Tipton

**Affiliations:** Purdue University

**Keywords:** homelessness, cervical cancer, screening, risk factors, prevention

## Abstract

**Objective:**

To estimate cervical cancer screening rates, prevalence of risk factors, and factors associated with being overdue for screening among people experiencing homelessness in two Indiana cities.

**Methods:**

Rapid assessment surveys were conducted at two large homelessness service agencies in Indianapolis and Lafayette, Indiana (November 2023 to November 2024). Participants were aged 21–69 years, assigned female at birth, and currently experiencing homelessness. Screening status was categorized as up-to-date (screened within 5 years) or overdue (more than 5 years or never screened). Descriptive statistics and multivariable logistic regression examined predictors of being overdue.

**Results:**

Among n = 212 participants, 35% were overdue and 49% had not been screened within 3 years. Prevalence of risk factors was high, including smoking (74%), sexual debut before age 18 (73%), and no HPV vaccination (75%). Older age and having experienced homelessness for 5 years or more were associated with higher odds of being overdue.

**Conclusions:**

Longer duration of homelessness significantly increased the likelihood of being overdue for cervical cancer screening, underscoring the cumulative disadvantage of chronic housing instability. Cervical cancer prevention is a critical unmet need among women experiencing homelessness, and findings highlight the importance of developing targeted interventions to improve screening access in this population.

## Introduction

Homelessness in the U.S. is a growing public health issue and humanitarian crisis, with a record-high 770,000 people experiencing homelessness (PEH) on a single night in 2024.^1^ PEH die nearly 30 years earlier than the average American,^2^ often from preventable illnesses.

Cancer is one of the most common causes of death among PEH;^3^ and cancer-related death rates in homeless adults are twice as high as in the general US adult population.^4^ Despite these statistics, these vulnerable populations are largely overlooked in cancer research.^5^ A 2018 systematic review revealed a dearth of data on cancer prevalence and cancer screening practices among PEH, with the limited available data indicating an overall higher incidence of cancer, higher rates of late-stage diagnosis, and higher mortality rates compared with the general population.^4^

A preventable and treatable disease, cervical cancer continues to be a marker of health disparity and limited healthcare access in the US. Screening is cost-effective and critical to successful cervical cancer management because human papillomavirus (HPV) infection, the primary etiologic agent, is asymptomatic, progression of precancerous lesions is slow, and treatment of advanced disease can be challenging and costly.^4^ However, homeless women have disproportionately lower rates of screening than the general population, and consequently higher cervical cancer incidence and mortality rates. The average rate of cervical cancer screening for U.S. women ages 21–65 years is 80.5%, with a mortality rate of 2.2 deaths per 100,000 women.^6^ Reported rates of cervical cancer screening among homeless women average 50%,^7-9^ with incidence rates up to fourfold higher than the housed population,^3^ and mortality rates as high as 7.2 deaths per 100,000 women. Furthermore, many homeless women who are screened report never learning of their results.^10^

In addition to disproportionate rates of underlying physical and mental health conditions, substance use disorders, and higher prevalence of key risk factors for cervical cancer, PEH also face unique, multilevel barriers to preventive healthcare. Socioeconomic deprivation, low health literacy levels, mistrust in healthcare providers due to stigma and discrimination, and difficult living conditions often disenfranchise them from health and social services.^11,12^ Even beyond structural barriers, homeless women in particular experience high rates of sexual violence and sexual trauma,^13-15^ often leading to discomfort with cervical cancer screening and delays or refusals of Pap tests.^14^ Homeless populations also report higher prevalence of key risk factors for cervical cancer, including smoking, poor diet, high-risk sexual behaviors and vulnerability to survival sex, alcohol/drug use, and sexually transmitted disease transmission.^16^

The few studies on cervical cancer among homeless populations in the U.S. are largely limited to California, New York, and Boston.^10,14,17-19^ An established multisectoral community-academic partnership in Indiana, which has been working together since 2020 to understand and address the diverse health needs of PEH in Indiana,^11,12,20-22^ identified cervical cancer as a priority area to understand and address in our own Midwestern context. Homelessness in Indiana has been steadily rising since 2021, with a record 6,285 PEH in 2024.^1^ Indiana last reported a cervical cancer incidence rate of 7.9 per 100,000 women, death rate of 2.3 per 100,000 women, and screening prevalence among women aged 21–65 years of 76%, all worse than national averages, and no data on these measures in homeless communities is available.^23^ The objective of this study is to estimate cervical cancer screening rates, risk factor prevalence, and factors associated with unscreened or underscreened individuals in two major homeless communities in Indiana, addressing a major paucity of data on this uniquely vulnerable population with excess and preventable cancer burden.

## Methods

### Study and instrument design

This community-engaged study was informed by an established multisectoral community-academic partnership addressing health needs among homeless populations in Indiana.^20^ A community advisory board, consisting of homelessness service providers, healthcare providers, community health workers, representatives of the Indiana Breast and Cervical Cancer Program (IN-BCCP), and a community member with lived experience of homelessness, was established specifically to guide this work. The advisory board reviewed and approved the study design and data collection instruments.

A rapid assessment survey was developed to document the prevalence of cervical cancer screening and other cervical cancer risk factors within this homeless population, as well as to identify characteristics (including age, race, ethnicity, gender identity, educational attainment, insurance status, and lifetime years of homelessness) of individuals who were overdue for screening (had never been screened in their lifetime or were last screened more than three to five years ago per national clinical guidelines. The U.S. Preventive Services Task Force (USPSTF) recommends screening for cervical cancer with the Pap test alone every 3 years in women aged 21 to 29 years. In women aged 30 to 65 years, the USPSTF recommends the Pap test alone every 3 years or HPV testing, with or without Pap co-testing, every 5 years. USPSTF guidelines also state that screening may be clinically indicated in women older than 65 with an inadequate or unknown screening history, therefore our eligibility includes women up to age 70. In our study, participants who reported being screened in the previous 5 years are considered up-to-date, and those who were never screened or last screened more than 5 years ago are considered overdue. Due to well-documented challenges in determining by self-report alone which type of cervical cancer screening test a woman received (i.e., a Pap smear, HPV test, or both),^24^ we additionally report participants who were last screened in the previous 3 to 5 years as possibly overdue.

To ensure accessibility across literacy levels, the rapid assessment survey was designed using plain language, administered orally, and limited to no more than 10 minutes. Survey questions were sourced from national data sets where applicable.^25,26^ Questions regarding cervical cancer risk factors were limited to those specified by the American Cancer Society.^27^

### Recruitment and data collection

Surveys were conducted at two major homelessness service agencies in Lafayette and Indianapolis, Indiana between November 2023 and November 2024. Indianapolis is the state’s capital and largest urban metropolitan area, which contrasts with Lafayette, a smaller metropolitan area surrounded by predominantly rural counties. Target enrollment was 200 participants (approximately 100 at each site). Eligibility was restricted to individuals currently experiencing homelessness, assigned female at birth, and aged 21 to 70 years. PEH were recruited passively through flyers/postcards containing information about the study and study team contact information, and announcements at the shelters.

Prior to participating, a study team member read the consent form aloud to the interested participant and provided them with a copy. The research team obtained verbal consent from each participant. Upon informed consent, the study team member read the survey questions aloud to the participant and recorded their answers electronically on password-protected iPad tablets or laptops through REDCap, a secure web application for building and managing online surveys and databases. No names, contact information, or identifying information were collected or stored. All participants were assigned a study ID at random.

Rapid assessment surveys took place at the two homeless shelters in a private location. Participants received a $10 gift card to a local grocery store upon completion of the survey in compensation for their time. This payment amount was deemed appropriate by community partners and commensurate with participant time. The study was reviewed and approved by the University Institutional Review Board, protocol: IRB-2023-940.

### Statistical analysis

Descriptive statistics were calculated to summarize the distribution of key study variables, including frequencies and percentages for categorical variables such as socio-demographic characteristics, lifetime duration of homelessness, cervical cancer risk factors, and screening status. Lifetime duration of homelessness is presented both as a continuous variable and also categorized as < 1 year, 1–2 years, 2–5 years, and > 5 years to reflect meaningful gradients in homelessness duration relevant to screening access. The < 1-year cutoff reflects the threshold commonly used by the U.S. Department of Housing and Urban Development (HUD), distinguishing short-term from longer-term (chronic) homelessness. Because our measure captured total lifetime homelessness rather than consecutive duration, we included a 1–2-year category to differentiate participants with moderate cumulative homelessness exposure from those with only brief or more prolonged periods of homelessness. The 2–5-year category represents sustained multi-year instability that is likely to affect continuity of care. Finally, > 5 years captures chronic, long-term homelessness and aligns with clinically meaningful cervical cancer screening intervals, as individuals unscreened for ≥ 5 years are considered overdue. Screening status was dichotomized as up-to-date (last screened in the past 5 years) or overdue (never screened or last screened more than 5 years ago). To examine bivariate associations between cervical cancer screening status and participant characteristics, chi-square tests were used for categorical variables, and independent-samples t-tests were employed for continuous variables.

Multivariable logistic regression models were used to identify predictors of being overdue for cervical cancer screening. Specifically, a stepwise model was employed to identify predictors of being overdue for cervical cancer screening. This approach allowed us to examine how different domains of variables contributed to the outcome in a structured and interpretable manner. In the first step, we included core sociodemographic factors, which represent baseline characteristics. In the second step, we added a key homelessness-specific variables, duration of lifetime homelessness, and current housing status to capture the unique experiences of the study population. By building the model sequentially, we were able to assess the homelessness-related factor beyond sociodemographic characteristics alone. This method highlights whether and how the distinct circumstances of PEH contribute independently to the likelihood of being overdue for cervical cancer screening.

Categorical variables were recoded, when necessary, to consolidate similar groups and small sample sizes. All analyses were limited to complete cases, as cases with missing data were excluded. Statistical analyses were performed using SPSS Statistics, Version 26.0 (IBM Corp., Armonk, NY).

## Results

Survey data were collected from n = 212 participants from two large homeless shelters in Indianapolis (n = 119) and Lafayette (n = 93), Indiana (Table 1). The mean age of respondents was 43.36 years, ranging from 21 to 69. Most participants identified as women (97%), non-Hispanic (94.3%), and white (55.2%), followed by Black (32.1%) and other racial identities (12.7%). Half reported being single/never married (50%). Participants had experienced an average of 36 months of lifetime homelessness, with over half (58.4%) having been homeless for more than 1 years and 21.5% for more than 5 years. At the time of the survey, 47.2% of participants experienced unsheltered homelessness. Approximately 1 in 4 participants (24.1%) had not completed high school, and the majority (59.9%) reported having no income. Most participants had public health insurance (81.6%), with very few reporting private insurance (2.8%), and the remainder (15.6%) uninsured. Half reported their health status as fair or poor (50.5%) and 44.3% reported having some kind of disability.

**Figure 1:**
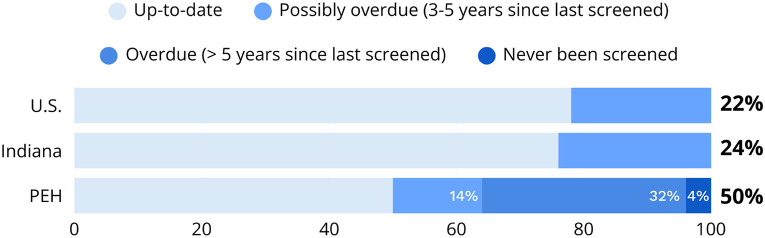
Cervical cancer screening rates. U.S.: national cervical cancer screening up-to-date rate.^23^ Indiana: state cervical cancer screening up-to-date rate.^23^ PEH: people experiencing homelessness (survey respondents), up to 50% possibly overdue (14% last screened in past 3-5 years, 32% last screened 5 years or more, 4 % never screened).

Over a third of participants (35.4%) were overdue for cervical cancer screening (never been screened or had not been screened in the previous 5 years or more) and another 14.6% had not been screened in the previous 3 years thus were possibly overdue (Fig. 1).

Participants overdue for screening were older (mean age = 46.61 years) compared to those who were up-to-date (mean age = 41.58 years, SD; p = 0.003). Those overdue had also spent more time homeless (mean = 46.10 months) compared to those up-to-date (mean = 30.86 months; p = 0.049). Among participants who had experienced over 5 years of homelessness, nearly half (48.9%) were overdue for screening, compared to 40.7% among those experiencing 2–5 years, 21.7% among those experiencing 1–2 years, and 27.6% among those experiencing less than 1 year of homelessness (p = 0.037). Race was significantly associated with screening status (p = 0.008), with a higher proportion of Black participants up-to-date on screening (79.4%) compared to white participants (57.3%). A statistically significant difference in screening status was also observed across locations (p = 0.019) as PEH in the Lafayette shelter were more likely to be overdue (44.1%) than those in the Indianapolis shelter (28.6%).

The prevalence of known risk factors for cervical cancer are shown in Table 2. Most participants had not been vaccinated against HPV (74.5%), most had a family history of cancer (76.9%), most had used birth control pills (68.9%), 41% had been previously diagnosed with a sexually transmitted infection including a small percentage (3.3%) with a known previous diagnosis of HPV, 74.1% had multiple sexual partners, and 73.1% had initiated sexual activity before age 18. The majority (81.1%) had carried a full-term pregnancy, and 41% had 3 or more pregnancies. Most participants smoked (73.6%), and more than half (54.2%) do not eat 2 or more cups of fruits and vegetables per day. HPV vaccination status was the only factor significantly associated with screening status: participants who had received the HPV vaccine were more likely to be up-to-date (77.8%) compared to those unvaccinated (60.1%; p = 0.019).

In the fully adjusted model (Step 2), older age was significantly associated with being overdue (OR = 1.066, 95% CI: 1.025–1.108, p = 0.001). Black participants had significantly lower odds of being overdue compared to white participants (OR = 0.285, 95% CI: 0.111–0.732, p = 0.009).

Duration of homelessness also emerged as a predictor of screening status. Participants who had been homeless 5 or more years were significantly more likely to be overdue (OR = 2.644, 95% CI: 1.051–6.652; p = 0.039) than those homeless for less than 1 year. No other variables were significantly associated with screening status in the adjusted model.

## Discussion

In this community-based rapid assessment of PEH in two Indiana cities, we found substantial gaps in cervical cancer prevention. Nearly half of participants had not been screened within the prior three years, and more than one-third were more than 5 years overdue. These rates are markedly worse than national and state averages, where approximately 22% and 24% of U.S. and Indiana women, respectively, are overdue for screening.^23^ These deficits occurred within a population reporting disproportionately high prevalence of cervical cancer risk factors compared to U.S. national averages, including smoking (74% compared with approximately 10% among women^28^), early sexual debut (73% compared with approximately 55%^29^), and extremely low HPV vaccination coverage (26% compared with approximately 57%^30^). Older participants and those with prolonged cumulative homelessness (5 years or more) had significantly higher odds of being overdue for cervical cancer screening.

Our results expand a small but growing body of literature documenting disparities in cervical cancer screening and risk factor prevalence among PEH. U.S.-based studies, primarily from Boston, New York, and California, report low screening uptake, low acceptance of Pap tests, and discomfort with pelvic examinations, particularly among women with histories of sexual trauma.^10,14,31-33^ This aligns with our own prior research highlighting that trauma, mistrust, stigma, and previous negative encounters with health systems often influence PEH engagement with healthcare services.^11,12,20-22^ International studies from the United Kingdom, Australia, France, and Canada similarly describe complex multilevel barriers, including socioeconomic deprivation, low health literacy, competing priorities, and logistical limitations such as transportation or documentation requirements.^18,34-37^ Our findings add new evidence from a Midwestern context, where homelessness has risen sharply in recent years,^1^ and where health system structures, rural-urban dynamics, and resource availability differ from coastal urban settings typically represented in the literature.

Chronic homelessness, defined in federal policy as 12 months or more of continuous or repeated episodes, has been linked to fragmented care and decreased participation in preventive health services. Our finding that women with 5 years or more of lifetime homelessness were more than twice as likely to be overdue aligns with international evidence showing that duration of homelessness is a key predictor of never having been screened.^37^ These findings highlight the cumulative disadvantage of prolonged housing instability as a structural barrier to cancer prevention.

The lower odds of being overdue among Black participants compared with white participants warrant further exploration. Prior studies have noted variation in screening coverage by race among PEH, often shaped by differences in engagement with safety-net clinics, community-based health programs, or prior reproductive health encounters. While our study cannot disentangle these pathways, the finding reinforces the importance of examining heterogeneity within homeless populations rather than assuming uniform risk or uniform access barriers.

The high prevalence of risk factors and striking disparities in screening status documented in this study underscore an urgent need for integrated, trauma-informed strategies to address both cervical cancer prevention and underlying determinants of health among PEH in the Midwest. Evidence suggests that trauma-informed pelvic examinations, patient navigation, and flexible and patient-centered screening models that meet the community where they are can significantly increase screening rates among PEH.

A Boston program showed improved screening participation after shifting from scheduled screening appointments to opportunistic, on-site screening embedded within routine clinical encounters.^38^ Patient navigation interventions have also been shown to mitigate multilevel barriers and to increase screening follow-through.^17^

Emerging cervical cancer screening modalities such as HPV self-sampling may offer additional promise in this population. Self-sampling has increased participation among underscreened women globally and is particularly relevant for women with histories of trauma or discomfort with speculum-based examinations.^39^ Ensuring public insurance coverage for self-sampling, and integrating it into mobile clinics, shelter-based care, and community outreach, could address several barriers simultaneously.

A key limitation of this study is that screening status and risk factors were self-reported, which may introduce recall and misclassification bias, particularly given the fragmented care and documentation challenges common among PEH. As a rapid assessment conducted at two shelters, findings may not generalize to all PEH or to those not accessing shelter services. Nonetheless, the study’s strengths include its community-engaged design, the input and guidance of a community advisory board, and representation from both urban and smaller metropolitan contexts in the Midwest.

## Conclusion

Cervical cancer is a preventable disease, yet its burden remains disproportionately concentrated among women with the least access to preventive care. Our findings highlight the need for multilevel and context-specific interventions to improve access to screening among PEH in Indiana. Future research should investigate the specific structural, behavioral, and trauma-related determinants of underscreening in Midwestern homeless communities and evaluate multicomponent interventions that combine trauma-informed care, patient navigation, opportunistic or mobile screening, and HPV self-sampling. Without targeted action, cervical cancer will remain a preventable contributor to excess morbidity and mortality among growing and uniquely vulnerable homeless populations.

## Supplementary Material

This is a list of supplementary files associated with this preprint. Click to download.

• Table2.docx

## Figures and Tables

**Table 1:** Participant demographics, overall and by screening status.

	Mean(SD)	Up-to-date	Overdue (> 5yrs)	p- value
**Demographics (n = 212)**				
Age	43.36(11.74)	41.58(11.96)	46.61(10.67)	0.003
Life Homeless Time (month) (n = 209)	36.18(53.44)	30.86(52.81)	46.10(53.54)	0.049
	Total n (%)	Up-to-date n (%)	Overdue n (%)	p- value
	N = 212	137 (64.6%)	75 (35.4%)	
**Location**				0.019
Lafayette	93 (43.9%)	52 (55.9%)	41 (44.1%)	
Indianapolis	119 (56.1%)	85 (71.4%)	34 (28.6%)	
Ethnicity				0.639
Non-Hispanic	200 (94.3%)	130 (65.0%)	70 (35.0%)	
Hispanic	12(5.7%)	7 (58.3%)	5 (41.7%)	
Race				0.008
White	117 (55.2%)	67 (57.3%)	50 (42.7%)	
Black	68 (32.1%)	54 (79.4%)	14 (20.6%)	
Others	27 (12.7%)	16 (59.3%)	11 (40.7%)	
Gender identity				0.915
Women	206 (97.2%)	133 (64.6%)	73 (35.4%)	
Others	6 (2.8%)	4 (66.7%)	2 (33.3%)	
Marital Status				0.117
Single/Unknown	106 (50.0%)	75 (70.8%)	31 (29.2%)	
Married/Partnership	29 (13.7%)	15 (51.7%)	14 (48.3%)	
Divorced/Widowed/Separated	77 (36.3%)	47 (61.0%)	30 (39.0%)	
Housing				0.700
Sheltered	99 (46.7%)	65 (65.7%)	34 (34.3%)	
Unsheltered	100 (47.2%)	65 (65.0%)	35 (35.0%)	
Couch Surfed	13 (6.1%)	7 (53.8%)	6 (46.2%)	
Life Homeless Time (month)				0.037
Life homeless time < 1 years (ref)	87 (41.6%)	63 (72.4%)	24 (27.6%)	
Life homeless time 1–2 years	23 (11.0%)	18 (78.3%)	5 (21.7%)	
Life homeless time 1–5 years	54 (25.8%)	32 (59.3%)	22 (40.7%)	
Life homeless time ≥ 5 years	45 (21.5%)	23 (51.1%)	22 (48.9%)	
Education				0.748
Completed high school or above	161 (75.9%)	105 (65.2%)	56 (34.8%)	
Did not complete high school	51 (24.1%)	32 (62.7%)	19 (37.3%)	
Income				0.983
Yes	85 (40.1%)	55 (64.7%)	30 (35.3%)	
No	127 (59.9%)	82 (64.6%)	45 (35.4%)	
Insurance				0.654
Private	6 (2.8%)	4 (66.7%)	2 (33.3%)	
No insurance	33 (15.6%)	19 (57.6%)	14 (42.4%)	
Public	173 (81.6%)	114 (65.9%)	59 (34.1%)	
Health Status				0.443
Excellent/Very Good	30 (14.2%)	22 (73.3%)	8 (26.7%)	
Good	75 (35.4%)	51 (68.0%)	24 (32.0%)	
Fair	70 (33.0%)	43 (61.4%)	27 (38.6%)	
Poor	37 (17.5%)	21 (56.8%)	16 (43.2%)	
Disability				0.614
No	118 (55.7%)	78 (66.1%)	40 (33.9%)	
Yes	94 (44.3%)	59 (62.8%)	35 (37.2%)	

**Table 3:** Stepwise multivariable logistic regression model for being overdue for cervical cancer screening (not screened in the last 5 years or more).

	Step 1			Step 2		
	Exp(B)	95% C.I.	Sig.	Exp(B)	95% C.I.	Sig.
Age	1.058	1.019–1.098	0.003	1.066	1.025–1.108	0.001
Race_White (Ref)						
Race_Black	0.237	0.095–0.595	0.002	0.285	0.111–0.732	0.009
Race_Others/Unknown	1.033	0.372–2.866	0.950	1.072	0.377–3.049	0.896
Ethnicity_Non-Hispanic (Ref)						
Ethnicity_Hispanic	1.691	0.417–6.855	0.462	1.312	0.303–5.683	0.717
GenderID_Women(Ref)						
GenderID_Others/Unknown	1.095	0.162–7.410	0.926	0.888	0.125–6.321	0.906
Marital Status_Single (Ref)						
Married/Partnership	1.934	0.647–5.781	0.238	2.047	0.664–6.305	0.212
Divorced/Widowed/Separated	0.580	0.254–1.324	0.196	0.552	0.232–1.311	0.178
Education_High school and above (Ref)						
Education_Below high school	0.900	0.406–1.993	0.794	0.979	0.428–2.237	0.959
Income_Yes (Ref)						
Income_No/Unknow	1.438	0.696–2.970	0.326	1.685	0.776–3.657	0.187
Insurance_Private (Ref)						
Insurance_No insurance/Unknown	2.949	0.285–30.485	0.364	2.296	0.225–23.465	0.483
Insurance_Public	1.578	0.175–14.217	0.684	1.205	0.135–10.773	0.867
Health Status Excellent/Very Good(Ref)						
Health Status_Good	1.571	0.497–4.970	0.442	1.542	0.464–5.128	0.480
Health Status_Fair	2.446	0.767–7.804	0.131	2.557	0.772–8.468	0.124
Health Status_Poor/Unknown	2.381	0.670–8.468	0.180	2.275	0.609–8.503	0.222
Disability_No (Ref)						
Disability_Yes	0.742	0.351–1.570	0.435	0.710	0.325–1.549	0.390
HPV Vaccine_Yes (Ref)						
HPV Vaccine_No/Unknown	1.890	0.806–4.434	0.143	1.839	0.758–4.466	0.178
Family History_No (Ref)						
Family History_Yes	1.100	0.488–2.478	0.818	1.239	0.529–2.904	0.621
Birth Control_No (Ref)						
Birth Control_Yes	0.750	0.349–1.611	0.460	0.635	0.286–1.409	0.264
IUD_No (Ref)						
IUD_new = Yes	0.904	0.380–2.151	0.819	0.829	0.336–2.046	0.684
STIs_No (Ref)						
STIs_All STIs*	0.675	0.326–1.396	0.289	0.674	0.312–1.454	0.314
> 1 Sexual Partner_No (Ref)						
> 1 Sexual Partner_Yes	0.539	0.237–1.223	0.139	0.552	0.238–1.278	0.165
< 18 First Sexual Intercourse_No (Ref)						
< 18 First Sexual Intercourse_Yes	2.414	0.971–6.001	0.058	2.428	0.954–6.181	0.063
Age > 20 first full-term pregnancy(Ref)						
Age < = 20 first full-term pregnancy	1.358	0.601–3.072	0.462	1.145	0.485–2.705	0.757
Full-term Pregnancies_0 (Ref)						
Full-term Pregnancies_1 to 2	1.211	0.415–3.533	0.726	1.298	0.439–3.837	0.637
Full-term Pregnancies_3	0.603	0.191–1.910	0.390	0.557	0.174–1.789	0.326
Smoke_No (Ref)						
Smoke_Current/Previously Smoked	0.948	0.414–2.168	0.899	0.847	0.361–1.988	0.703
>=2 Cups of Fruit and Veg /Day (Ref)						
< 2 Cups of Fruit and Veg/Day	1.364	0.687–2.708	0.375	1.340	0.66–2.721	0.418
Housing Status_Sheltered (Ref)						
Housing Status_Unsheltered				0.880	0.420–1.845	0.735
Housing Status_Others				1.386	0.276–6.954	0.692
Life Homeless Time_<1 Year (Ref)						
Life Homeless Time_1–2 Years				0.626	0.164–2.397	0.494
Life Homeless Time_2–5 Years				2.046	0.860–4.870	0.106
Life Homeless Time_>5 Years				2.644	1.051–6.652	0.039

* Other STI and HPV were grouped in regression (All STIs)

## Data Availability

Data is available upon request from natalia@purdue.edu.

## References

[1] The 2024 (2024) Annual Homelessness Assessment Report (AHAR to Congress) Part 1: Point-In-Time Estimates of Homelessness, December

[2] Homeless-Mortality-Toolkit -FULL-FINAL, Accessed (2025) December 15. https://nhchc.org/wp-content/uploads/2020/12/Homeless-Mortality-Toolkit-FULL-FINAL.pdf

[3] Baggett TP, Chang Y, Porneala BC, Bharel M, Singer DE, Rigotti NA (2015) Disparities in Cancer Incidence, Stage, and Mortality at Boston Health Care for the Homeless Program. Am J Prev Med 49(5):694–702. 10.1016/j.amepre.2015.03.038 26143955 PMC4615271

[4] Asgary R (2018) Cancer screening in the homeless population. Lancet Oncol 19(7):e344–e350. 10.1016/S1470-2045(18)30200-6 30084381

[5] Kushel MB, Vittinghoff E, Haas JS (2001) Factors Associated With the Health Care Utilization of Homeless Persons. JAMA 285(2):200–206. 10.1001/jama.285.2.200 11176814

[6] Increase the proportion of females who get screened for cervical cancer — C–09 - Healthy People 2030 ∣ health.gov. Accessed May 17, 2022. https://health.gov/healthypeople/objectives-and-data/browse-objectives/cancer/increase-proportion-females-who-get-screened-cervical-cancer-c-09

[7] Chau S, Chin M, Chang J (2002) Cancer Risk Behaviors and Screening Rates Among Homeless Adults in Los Angeles County. Cancer Epidemiol Biomarkers Prev 11(5):431–438 12010856

[8] Long HL, Tulsky JP, Chambers DB (1998) Cancer Screening in Homeless Women: Attitudes and Behaviors. J Health Care Poor Underserved 9(3):276–292. 10.1353/hpu.2010.0070 10073209

[9] Diamant AL, Brook RH, Fink A, Gelberg L (2002) Use of Preventive Services in a Population of Very Low-Income Women. J Health Care Poor Underserved 13(2):151–163. 10.1353/hpu.2010.0552 12017906

[10] Asgary R, Alcabes A, Feldman R (2016) Cervical cancer screening among homeless women of New York City shelters. Matern Child Health J 20(6):1143–1150. 10.1007/s10995-015-1900-1 26649876 PMC4873360

[11] Rodriguez NM, Lahey AM, MacNeill JJ, Martinez RG, Teo NE, Ruiz Y (2021) Homelessness during COVID-19: Challenges, Responses, and Lessons Learned from Homeless Service Providers in Tippecanoe County, Indiana. In Review; 10.21203/rs.3.rs-583647/v1 PMC8432956 34507565

[12] Rodriguez NM, Martinez RG, Ziolkowski R, Tolliver C, Young H, Ruiz Y (2022) COVID knocked me straight into the dirt: perspectives from people experiencing homelessness on the impacts of the COVID-19 pandemic. BMC Public Health 22(1):1327. 10.1186/s12889-022-13748-y 35820879 PMC9275174

[13] Breton M, Bunston T (1992) Physical and Sexual Violence in the Lives of Homeless Women. Can J Commun Ment Health 11(1):29–44. 10.7870/cjcmh-1992-0003

[14] Kohler RE, Roncarati JS, Aguiar A (2021) Trauma and cervical cancer screening among women experiencing homelessness: A call for trauma-informed care. Womens Health 17:174550652110292. 10.1177/17455065211029238 PMC8264729 34225506

[15] Sundin EC, Baguley T (2015) Prevalence of childhood abuse among people who are homeless in Western countries: a systematic review and meta-analysis. Soc Psychiatry Psychiatr Epidemiol 50(2):183–194. 10.1007/s00127-014-0937-6 25178273

[16] Caccamo A, Kachur R, Williams SP (2017) Narrative Review: Sexually Transmitted Diseases and Homeless Youth—What Do We Know About Sexually Transmitted Disease Prevalence and Risk? Sex Transm Dis 44(8):466–476. 10.1097/OLQ.0000000000000633 28703725 PMC5778439

[17] Asgary R, Naderi R, Wisnivesky J (2017) Opt-Out Patient Navigation to Improve Breast and Cervical Cancer Screening Among Homeless Women. J Womens Health 26(9):999–1003. 10.1089/jwh.2016.6066 28103125

[18] Moravac CC (2018) Reflections of Homeless Women and Women with Mental Health Challenges on Breast and Cervical Cancer Screening Decisions: Power, Trust, and Communication with Care Providers. Front Public Health 6:30. 10.3389/fpubh.2018.00030 29600243 PMC5863503

[19] Pieters HC, Wiley DJ (2013) Decision-making about cervical cancer screening methods by homeless women. J Natl Black Nurses Assoc 24(1):9–15 24218868

[20] Rodriguez NM, Ziolkowski RA, Hicks J Infectious Disease Preparedness for Homeless Populations: Recommendations from a Community-Academic Partnership. Prog COMMUNITY Health Partnersh Res Educ ACTION PCHP Forthcom. https://preprint.press.jhu.edu/pchp/preprints/infectious-disease-preparedness-homeless-populations-recommendations-community-academic 38661832

[21] Rodriguez NM, Cromer R, Martinez RG, Ruiz Y (2022) Impact of COVID-19 on People Experiencing Homelessness: A Call for Critical Accountability. Am J Public Health 112(6):828–831. 10.2105/AJPH.2022.306768 35446605 PMC9137024

[22] Ziolkowski RA, Balian L, Sridhar S, Rodriguez NM (2024) Improving uptake of COVID-19 testing and vaccination in a homeless population: mixed-methods evaluation of community health worker-led education in a shelter. BMJ Open 14(12):e087134. 10.1136/bmjopen-2024-087134 PMC11647339 39658289

[23] USCS Data Visualizations. Accessed December 15 (2025) https://gis.cdc.gov/grasp/USCS/DataViz.html

[24] Cervical Cancer Screening. Accessed December 15 (2025) https://progressreport.cancer.gov/detection/cervical_cancer

[25] Health Information National Trends Survey ∣ HINTS. Accessed December 15 (2025) https://hints.cancer.gov/

[26] Behavioral Risk Factor Surveillance System. September 12 (2025) Accessed December 15, 2025. https://www.cdc.gov/brfss/index.html

[27] Cervical Cancer Risk Factors ∣ Risk Factors for Cervical Cancer. Accessed December 15 (2025) https://www.cancer.org/cancer/types/cervical-cancer/causes-risks-prevention/risk-factors.html

[28] CDCTobaccoFree (2024) Burden of Tobacco Use in the U.S. Centers for Disease Control and Prevention. October 8, Accessed December 15, 2025. https://www.cdc.gov/tobacco/campaign/tips/resources/data/cigarette-smoking-in-united-states.html

[29] National Health Statistics Reports (2017) Number 104, June 22

[30] HPV Vaccination. Accessed December 15 (2025) https://progressreport.cancer.gov/prevention/hpv_immunization

[31] Jeleff M, Haider S, Schiffler T (2024) Cancer risk factors and access to cancer prevention services for people experiencing homelessness. Lancet Public Health 9(2):e128–e146. 10.1016/S2468-2667(23)00298-0 38307679

[32] Bharel M, Casey C, Wittenberg E (2009) Disparities in Cancer Screening: Acceptance of Pap Smears among Homeless Women. J Womens Health 18(12):2011–2016. 10.1089/jwh.2008.1111 20044864

[33] Galvin AM, Akpan IN, Garg A, Cuccaro PM, Thompson EL, Santa Maria DM (2025) Human Papillomavirus–Related Cancer Prevention Among People Experiencing Housing Instability: A Systematic Review. Sex Transm Dis 52(7):381. 10.1097/OLQ.0000000000002159 40085435

[34] Hawkins KE, Gourlay K, Cuschieri K (2024) Challenges for cervical screening in people experiencing homelessness. BMJ Sex Reprod Health 50(2):150–151. 10.1136/bmjsrh-2023-202023 38182271

[35] Lawrie K, Charow R, Giuliani M, Papadakos J, Homelessness (2020) Cancer and Health Literacy: A Scoping Review. J Health Care Poor Underserved 31(1):81–104 32037319 10.1353/hpu.2020.0010

[36] Lovell RC, Botfield JR, Cheng Y, Tilley DM, Fazio A, Estoesta J (2020) Promoting cervical screening among women experiencing homelessness and socio-economic disadvantage in Sydney. Health Promot J Austr 31(3):357–368. 10.1002/hpja.322 31978250

[37] Vuillermoz C, Vandentorren S, Roze M, Rondet C, Chauvin P (2017) Cervical cancer screening among homeless women in the Greater Paris Area (France): results of the ENFAMS survey. Eur J Cancer Prev 26(3):240. 10.1097/CEJ.0000000000000225 26895575

[38] Bharel M, Santiago ER, Forgione SN, León CK, Weinreb L (2015) Eliminating Health Disparities: Innovative Methods to Improve Cervical Cancer Screening in a Medically Underserved Population. Am J Public Health 105(Suppl 3):S438–S442. 10.2105/AJPH.2014.302417 25905832 PMC4455514

[39] Nelson EJ, Maynard BR, Loux T, Fatla J, Gordon R, Arnold LD (2017) The acceptability of self-sampled screening for HPV DNA: a systematic review and meta-analysis. Sex Transm Infect 93(1):56–61. 10.1136/sextrans-2016-052609 28100761

